# Hospital and Institutional News

**Published:** 1921-03-26

**Authors:** 


### HOSPITAL AND INSTITUTIONAL NEWS.

### DEATH OF MR. HENRY BONHAM-CARTER.

As we go to press we learn with deep regret of the death of Mr. Henry Bonham-Carter, who passed away on March 22 at the great age of ninety-four. He was the first cousin of Florence Nightingale, and with her and Mrs. Wardrope, the great matron of St. Thomas's Hospital and the Nightingale Training School, was responsible for the rules and regulations for training nurses which have made the term Nightingale nurse the synonym for the highest and best type of nurse. It is interesting to recall that the first secretary to the Nightingale Fund was Arthur Hugh Clough, the poet, Mr. Henry Bonham-Carter succeeding him as secretary *pro tem.* in June 1861; this appointment was made permanent in January 1862, and from that date till his retirement in 1914 Mr. Bonham-Carter devoted his untiring ability to the affairs of the Fund and the School, which prospered greatly. He served on the House Committee of St. Thomas's Hospital for many years, and attended regularly every week. When the Metropolitan Nursing Association was founded in 1875 he joined the Council, and later served on the Council of the Queen Victoria Jubilee Institute. Referring to Mr. Bonham-Carter's great services to the Nightingale Fund and School on his retirement in 1914, after fifty-three years of office, the Fund's report says:—"During that period he has seen the whole movement for training nurses and improving their status initiated and developed, and with the constant help of Miss Nightingale, with whom he was intimately associated and in frequent communication, and of the able women who successively acted as matron of St. Thomas's Hospital, he took a large part in originally planning out what had to be done and in subsequently attending to the many details inseparable from the management of the Training School." With Mr. Bonham-Carter disappears, we believe, the last living link with Miss Nightingale, in the development of whose ideals he has been so largely concerned.

### SCOTTISH BOARD OF HEALTH.

Dr. Norman Walker, Edinburgh, has been appointed Chairman of the Scottish Board of Health Consultative Council on Medical and Allied Services in succession to Principal Sir Donald MacAlister, K.C.B.; and Dr. John Yule Mackay, Principal of University College, Dundee, has been appointed Vice-Chairman in succession to Dr. Walker. While relinquishing the Chairmanship of the Council, Sir Donald MacAlister will continue to give his services as a member of the Council.

### THE SATURDAY FUND'S EXAMPLE.

The current month's issue of the Metropolitan Hospital Saturday Fund *Journal* is mainly concerned with the details of its own work, which the now customary short story and the verse agreeably garnish. But Mr. Inman, as we suppose, begins with a useful leading article, called "The First £100,000," which again records facts whereof we can hardly be made too fully aware. First, the aim last year of the Fund's officials to reach £100,000 was realised; in fact, £106,000 was contributed, which meant an increase of £31,000 over the sum raised in 1919. That is very encouraging, for it was raised by an organisation which in its nature can be permanent, and was not the fruits of a soon exhausted spirit. For this reason, now that the scheme is on its legs, the Fund hopes to raise the total to £150,000 this year. It is not an unreasonable hope, and we watch Mr. Inman's efforts in this direction with the warmest interest. As doubtless he is aware, much depends upon the success of his extended scheme. The voluntary hospitals have still crises to face, though on the whole they have survived the peace very well indeed. London will always remain the weak spot until the leaven of organised contributions can permeate it. As The Hospital pointed out a year ago, there is no reason why, when the population is considered, £300,000 a year should not be raised eventually. The Fund's quiet but steady progress is the best earnest of that future result. We cannot be too grateful for it.

### SATURDAY AND SUNDAY FUNDS IN NORWICH.

At the annual meeting of the Norwich Hospitals and Medical Institutions Sunday and Saturday Funds at Norwich it was announced that the amount for distribution was £6,417, as against £2,912 in the previous year. This is excellent, and another illustration of the value of the small contribution when properly organised. Although in the churches and schools collections showed a larger yield, it was in the factories and workshops, under a contributory scheme, that the main part of the fund was raised. One of the speakers said that it is clear that some organised movement such as that at Norwich is inevitable if the hospitals are to be retained on a voluntary basis, and he was confident of the result, as hospitals stand for efficiency, cleanliness, and health, as well as for mercy, compassion, and brotherhood.

### ADOPT A HOSPITAL!

Our contemporary the *Daily Express* has done excellent service for Metropolitan hospitals with its Adopt-a-Hospital scheme, started last autumn. We understand that eleven banks have taken up the adoption scheme, which entails an organised collection amongst the staff for a particular hospital. The Royal Free has won the sympathy of the Standard Bank of South Africa, a member's contribution being 1s. a month. Upon inquiry, however, at one of the banks mentioned a hospital Secretary found that the manager was unaware of the bank's alleged foster-parenthood, but, on being reminded that it is a wise father that knows his own child, came into line.

### DISPENSARIES AND UNEMPLOYMENT.

The expected tide of unemployment has affected hospitals more than the general public is aware, and the dispensaries, perhaps, were the first institutions to feel it. For example, last year the number attending the old dispensaries at Liverpool was 26,590—about 1,000 more than in 1919—but what remains to be explained is that the number in 1918 was even higher.

### HOSPITAL MEDICAL APPOINTMENTS.

Appointment of honorary medical officers to hospital vacancies is the subject of a correspondence in one of the West-Country papers, and it is clear that it is thought by some that the present method is not satisfactory. One writer deplores the fact that in practice the honorary staff choose their own colleagues, which shuts out anybody who is not acceptable to the controlling personalities of the staff. "This deplorable state of affairs has," he explains, "existed for many years in many hospitals, and should, in the public interest, be put a stop to." His remedy is to open the beds to every practising surgeon, recognised by the profession as such, exactly as obtains in a nursing home. All systems have their advantages and disadvantages, and what may apply very suitably to a teaching hospital in a large city would not be appropriate to the case of a small general hospital. The whole question, which is sure to be qualified later by the increase of the paying-patient system, is a most interesting one at the present time, and we shall be glad to hear the views of our readers upon it.

### THE MIDDLE CLASSES UNION AND HOSPITALS.

We are glad to note that the Middle Classes Union is not merely protesting against rates and taxes and trying by organisation to protect the "black coat," but is also adopting a constructive policy. This latter is more sure of immediate success. The Union, of which Dr. W. B. Bennett is Chairman, apparently has approached the West Derby Guardians, already interested in middle-class patients, who, after inquiry, declare that "no burden will be added to the rates" if paying patients are admitted to the Mill Road Auxiliary Hospital at a fee of three guineas a week. The Guardians expect the scheme to be self-supporting, and free from anything which might prejudice this deserving class of patient against it. As The Hospital has already noted, Manchester and Liverpool are neck-and-neck in the same endeavour. Liverpool, we fancy, first announced the plan, but Manchester, we think, was the first to put it into practice. At all events, on March 31 a scheme will be tried for the benefit of paying patients residing in the West Derby Union; they are to be admissible either on their own application or on that of their medical practitioner, and in both cases, of course, subject to the discretion of the medical superintendent. Acute medical or surgical cases will be received at Mill Road Infirmary; no special inquiries or case papers will be required for them; patients (we are glad to add, of course) may wear their own clothes. They can be visited, without formality, every day except Sunday between 2 and 4 p.m., and their own doctors have the right to consult with the infirmary staff, though the responsibility for that must rest with the medical superintendent. A photograph of one of the wards has been published, and it should encourage patients to enter it. We are sure that the Union has done wisely in this matter. It is badly equipped for opposition to hostile bodies, because a trade union, in this sense, can only exist when its members all have the same income. But on the better and constructive side it can dominate, and we congratulate it on this effort.

### MISUNDERSTOOD.

A correspondent draws our attention to a singular advertisement which appears in the current number of the *Wandsworth Boro' News*. The advertisement is as follows: "Notice.—If the persons are found who are spreading false reports regarding the illness of Mrs. Blank, of No. Blank, Blank Street, they will be prosecuted.—Her Doctor." It is quite likely that the medical man in question is a good-natured practitioner who is also a friend of his patients, and he may have said a few comforting words which have led the patient or her friends to publish a warning that may or may not be necessary. In any case, such an advertisement is quite exceptional. General practitioners are not well advised in identifying themselves with local tittle-tattle as a general rule. This may possibly be one of the exceptional cases.

### PAYING PATIENTS IN INFIRMARIES.

The Minister of Health is keenly watching all schemes of Guardians for the reception of paying patients, and the latest inquiry he has made is at Barnet. As Dr. Addison learned at Bradford, a scheme for admitting persons other than the sick poor to the Wellhouse Hospital appears "to raise very important principles, medical and other," and he wants to know exactly what is proposed and if any steps have already been taken.

### THE HOSPITAL GUILD IN BIRMINGHAM.

With a debt for 1920 of £20,500, the Queen's Hospital, Birmingham, is in need of a gleam of hope, and this seems to be provided by the action of two firms, each of which made up the annual contribution of their employees from 8s. 8d. to one pound per head. That is a fine lead and one which we hope may prove infectious. For £10,000 a year more is necessary to meet the working expenses—apart from the new medical out-patient department and a modern operating-theatre. We observe, however, that more than £11,000 was received from donations, that the Saturday Fund's contribution showed an increase of £2,250, and the Sunday Fund also was £500 to the good. Still more encouraging is the news that the Hospital Guild, working under the Hospital Council, made a good start. The things to do seem to be to extend the Guild, and to try to persuade other firms to follow, in appropriate degrees, the generous lead given by the two mentioned above. On the whole, in short, the signs of growth are promising, because they may be made permanent, whereas legacies, though convenient, are only desperate remedies.

### A CHAIRMAN AND APPROVED SOCIETIES.

Mr. Roger Beck, chairman respectively of the Swansea Hospital Board and the Finance Committee, has given to the *South Wales Weekly Echo* his views on the proposal of the Committee of the London Hospital not to treat insured persons unless their approved societies support the institution. He is reported to have said: "I would not think of introducing the question here. The employees of the whole district of Swansea have done so magnificently, according to their means, for the Swansea Hospital that I would not think of damping their enthusiasm or restricting in such a way their free use of the hospital." But, he went on, "from my acquaintance with University College Hospital, London, and some other hospitals in manufacturing centres, I am astonished at the inadequate contributions received from employees." Is not this due to want of organisation, though organisation is far easier in a hospital used by a number of works with their respective staffs than to a hospital serving a part of a great city like London? Since 1916 the employees' contributions at Swansea have risen from £9,000 to £22,000, an increase which Mr. Beck believes to be unique. But because London is a city of amorphous industries and retail trades Mr. Beck agrees that organisation is more difficult. It is, however, none the less necessary. The hospitals' claims upon the surplus funds of approved societies are virtually unchallengeable, but these societies have hitherto been so indifferent to these claims that to be asked to meet them now is a shock, though one which the societies have largely brought upon themselves.

### NEVER IN DEBT.

If all hospitals could manage things as well as, or were fortunate as, the Poplar Hospital, the hospital problem would be solved. Sir Joseph Brookland, presiding at the council meeting of subscribers, announced that the boast of never being in debt held good for the past year. The building work is to be carried out at a cost of £54,000, and towards this £42,000 has been received.

### THE BOMBARDMENT OF CAMERON HOSPITAL.

The Cameron Hospital at West Hartlepool had the unenviable distinction of being bombarded during the war; and the Committee has long hoped to recognise the services of the staff by presenting its members, who were working under the bombardment, with commemorative medals. Unfortunately, and doubtless with some excuse, delay has hitherto prevented the fulfilment of this natural wish; and now the Sub-Committee in charge of the proposal reports that the then staff is scattered, that time in other ways has made the presentation difficult; and, finally, that the hospital is now so poor (it has an overdraft of £650) that the expense incurred would not be warrantable. We venture to brush aside all the difficulties, except that of finance, and this surely some friend of the hospital who appreciates heroism could be found to overcome. It seems, indeed, almost a case for the consideration of the Carnegie Fund Trustees, since the staff showed its bravery as a non-combatant body. The *esprit de corps* shown at the time deserves recognition: absent members can be traced where there is a will to trace them; and the trouble involved would be the best of recognition for the bravery it is desired to commemorate. We hope that Mr. Brankston, in spite of his financial anxieties, will weigh these arguments as they deserve, and still do his best to find a friend to provide the relatively small sum needed. Its gift indeed might encourage interest in the hospital and other help, and so, in fact, lead to the end of the general financial trouble.

### MR. E. J. THOMAS'S CRITICISM.

The Birmingham Ear and Throat Hospital, which has an alarmingly long waiting list, is plainly inadequate at present to meet the needs of the Midlands. There are various proposals to remedy this. Mr. Charles Elkin, Chairman of the Hospital Committee, appears to favour an ambitious scheme to remove all the special hospitals in Birmingham into the country, to unite them in one institution there under one control, but of course with separate units or departments. The realisation of such a scheme appears too remote to Mr. E. J. Thomas, who represents the Birmingham Hospital Saturday Committee. He declares, with warmth, that while hundreds are waiting for admission to such institutions as the Ear and Throat Hospital, empty beds are lying idle in the hospital in Dudley Road. He calls for immediate action. But do not the means exist for this? Birmingham has now a Hospital Council. The Guardians, surely, have already favoured co-ordination there? Why, then, does not Mr. Thomas, who carries weight because of the Fund for which he speaks, get this question discussed by both bodies?

### A FRIENDLY SOCIETY MEMBERS' SUGGESTION.

Mr. Ernest Rumbelow, of Maidstone, who has already contributed ideas and work for the neighbouring hospital, has made another suggestion worthy of notice. Understanding, he says, that "the results of the valuation of the State section of the Manchester Unity of Oddfellows will shortly be declared, and that the surplus will be distributed to institutions approved by the Ministry of Health," Mr. Rumbelow suggests to Oddfellows (he has been one himself, he writes, for twenty-four years) that they should remember the needs of the Kent County Ophthalmic Hospital Extension Fund, and, in effect, incite the various lodges to which they belong to make an annual grant from the surplus available. If members of approved societies could do the same, for the hospitals at which they may be patients, the pressure of public opinion would solve the question from within. We suggest that every hospital should get a petition signed by such patients, or, better still, induce them to frame one.

### ST. THOMAS'S APPEAL.

As a specimen of skilled advertising the sheet of interesting photographs of work at St. Thomas's Hospital in the weekly edition of *The Times* ranks high. The weighing and feeding of very young infants (689 babies were born in the Maternity ward last year) make telling pictures, not the least pleasing feature of which are the smiling nurses in attendance.

